# Insights on the Molecular Behavior of Polypropylene in the Process of Ultrasonic Injection Molding

**DOI:** 10.3390/polym13224010

**Published:** 2021-11-19

**Authors:** Jackeline Iturbe-Ek, Alan O. Sustaita, Diego Aguilar-Viches, José Manuel Mata-Padilla, Carlos A. Ávila-Orta, Luis E. Elizalde, Alex Elías-Zúñiga, Luis Marcelo Lozano

**Affiliations:** 1 Escuela de Ingeniería y Ciencias, Tecnologico de Monterrey, Av. Eugenio Garza Sada 2501, Monterrey 64849, Mexico; jackeline.iturbe.ek@tec.mx (J.I.-E.); daviches1991@gmail.com (D.A.-V.); aelias@tec.mx (A.E.-Z.); 2 CONACyT–Centro de Investigación en Química Aplicada, Blvd. Enrique Reyna 140, Saltillo 25294, Mexico; jose.mata@ciqa.edu.mx; 3 Centro de Investigación en Química Aplicada, Blvd. Enrique Reyna 140, Saltillo 25294, Mexico; carlos.avila@ciqa.edu.mx (C.A.Á.-O.); luis.elizalde@ciqa.edu.mx (L.E.E.)

**Keywords:** ultrasonic injection molding, polypropylene, product miniaturization, molecular characterization

## Abstract

Product miniaturization is a constant trend in industries that demand ever-smaller products that can be mass produced while maintaining high precision dimensions in the final pieces. Ultrasonic micro injection molding (UMIM) technology has emerged as a polymer processing technique capable of achieving the mass production of polymeric parts with micro-features, while still assuring replicability, repeatability, and high precision, contrary to the capabilities of conventional processing technologies of polymers. In this study, it is shown that the variation of parameters during the UMIM process, such as the amplitude of the ultrasound waves and the processing time, lead to significant modification on the molecular structure of the polymer. The variation of both the amplitude and processing time contribute to chain scission; however, the processing time is a more relevant factor for this effect as it is capable of achieving a greater chain scission in different areas of the same specimen. Further, the presence of polymorphism within the samples produced by UMIM is demonstrated. Similarly to conventional processes, the UMIM technique leads to some degree of chain orientation, despite the fact that it is carried out in a relatively small time and space. The results presented here aim to contribute to the optimization of the use of the UMIM process for the manufacture of polymeric micro parts.

## 1. Introduction

Today, the use of polymers in industries such as information technology (IT), biomedical, automotive, telecommunications, and aerospace is increasing because the properties offered by these materials, such as low cost and weight and resistance to corrosion, have become crucial. Furthermore, product miniaturization is a constant trend in many of these industries, which demand ever-smaller products that can be mass produced while preserving a high precision in the final pieces. This opens the opportunity for new manufacturing technologies that guarantee the requirements mentioned above, and through which the broadest possible variety of polymers can be processed, in order to provide a range of solutions to the challenges that such industries face every day.

Conventional processing technologies of polymers, such as extrusion, injection molding, compression molding, or blow molding, have been primarily used for the mass production of daily use commodities. However, such processes are no longer suitable for manufacturing small products with a dimensional precision in the micro-scale, or with a total mass ranging between 0.1 and 0.001 g. In this regard, micro-sized products based on polymers have potential applications as micromechanical structures, micro gear wheels, and in the medical, optical, and electronic industries, to name a few. Recently, ultrasonic micro injection molding (UMIM) technology has emerged as a polymer processing technique capable of overcoming the disadvantages presented by conventional methods and thus achieving mass production of polymeric parts with micro-features while assuring replicability, repeatability, and high precision, which are crucial in any industry.

In UMIM, polymeric micro parts are produced after the raw material is melted by the energy applied by ultrasonic vibration with frequencies of 30 KHz. The general overview of the process can be reviewed in detail elsewhere [1,2], and is briefly described as follows. The raw polymer in the form of pellets is firstly placed in the plasticization chamber. Then, the process starts and a sonotrode, which is the element that delivers the ultrasonic vibration to the material and is initially placed away from the polymer, moves in the direction of the pellets until it reaches them. At this point, it begins to vibrate as it continues its displacement, causing the material to melt. After a short time (in the range of a few seconds), the melted polymer is injected into a small cavity (mold) that has the geometry of the desired final part.

In essence, ultrasonic energy is converted to thermal energy that increases the temperature to a level sufficient to melt the polymer. According to Michaeli et al. [3], this ultrasonic energy produces the polymer plasticization mainly via two mechanisms: (i) the internal friction of the material, which is a factor related to material damping properties, and (ii) the friction caused by the relative movement between the pellets. This combination increases the local temperature until the polymer melts [4].

The effects of ultrasound on polymers can be physical and chemical, both contributing to reducing the viscosity of the polymer significantly. The primary chemical effects have mainly been associated with the cavitation phenomenon, which involves the nucleation, growth, and collapse of microbubbles, whose violent collapse induces a large amount of shear force on the polymer chains, causing rapid unwinding and, finally, their scission [5]. However, this mechanism has the limitation that it is mostly valid for fluids with Newtonian behavior, such as water or even polymers in solution. However, in non-Newtonian fluids such as molten polymers, the cavitation mechanism is limited due to the viscoelastic characteristics of polymeric matrices. The formation of bubbles is significantly hindered, causing only the few bubbles that could be formed to implode less violently compared to those in a Newtonian fluid such as water, which makes the impact of the liquid jet at the boundary very small or even zero [6]. 

Espinoza-González [7] proposed a mechanism based on mechanochemistry to explain the physical and chemical effects of ultrasound on polymeric matrices. This mechanochemical mechanism is based mainly on the deformation or tension that chemical bonds experience during the vibration movement caused by ultrasonic waves. This vibration movement causes the appearance of different fatigue points along the polymer chain, producing the greatest deformation between the links of the polymer chain and reducing the dissociation energy of the links. This effect can lead to the activation of multiple reaction mechanisms, degradation, or even chain extension, depending on the polymer type. Polymer chain scission occurs more rapidly for polymers with higher molecular weights, and this polymer cleavage progresses towards those with lower masses until a plateau is reached, implying no further polymer chain scission.

To date, UMIM technology has been demonstrated to be a suitable technique to process a wide range of thermoplastics, ranging from commodity polymers, such as polypropylene (PP) [8,9] and polyamide (PA) [4], to some engineering and biocompatible polymers, such as ultra-high molecular weight polyethylene (UHMWPE) [1], polyoxymethylene (POM) [3], and polylactic acid (PLA) [10], and reaching some high-performance engineering polymers such as poly (ether ether ketone) (PEEK) [11], and even polymer composites [2]. Many of the works referred to above were focused on performing a study of the optimal parameters of the UMIM technique to process each material, which is understandable and very useful for a newly emerging polymer processing technology. However, there is a lack of studies that delve into what happens with polymers at the molecular level in UMIM technology, in order to achieve a better understanding of the effect of ultrasound in the fabricated polymer micro parts, and to know in what way and to what extent UMIM technology can be exploited.

Hence, in this work, different characterization techniques were used to analyze the molecular structure of polypropylene specimens fabricated by UMIM after varying two parameters: the amplitude of ultrasound waves and the processing time. Just as with conventional polymer processing technologies, where it is possible to determine the effect that certain parameters such as temperature, residence time, and cooling rate will have on the final product, it is of great importance to know the effect of the parameters that are adjustable in the UMIM process on the fabricated part. However, given that this technology is mainly attractive for the manufacture of micro parts, the study at a molecular level of the manufactured parts becomes more relevant compared to conventional processes, and this is precisely what was sought in this work.

## 2. Materials and Methods

### 2.1. Materials

An isotactic homopolymer of polypropylene (iPP) with high crystallinity was used in this study (INDELPRO, Profax PL338). According to the material’s datasheet, its density is 0.9 g/cm^3^ and the melt flow rate (MFR) is 3 dg/min. This polymer is designed for extrusion processes, and its typical applications are those where high mechanical properties and high temperatures are required. 

### 2.2. Ultrasonic Micro-Injection Molding

**Figure 1:**
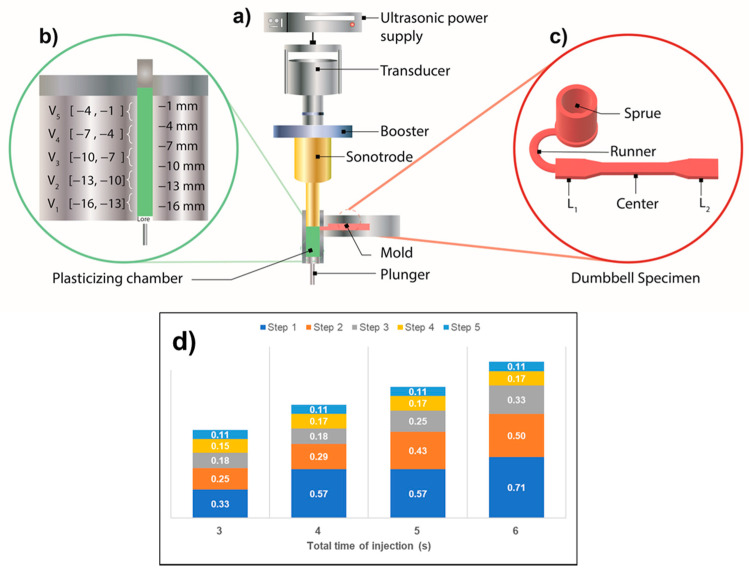
(**a**) Scheme of the UMIM machine; (**b**) injection distance in the plasticizer chamber, where the distances of the five steps are described; (**c**) dumbbell-shaped specimen obtained at the end of the UMIM process using a mold with the corresponding shape; and (**d**) bar graph showing in detail the inverse of the plunger displacement speed for each of the times selected in this study.

Dumbbell-shaped specimens with micro-features were obtained using an ultrasound microinjection molding machine (Sonorus 1G^®^, fabricated by Ultrasion S.L.), composed of four main parts: an ultrasound generator, a transducer, an acoustic unit, and a plunger. The detailed description of the machine can be seen in Figure 1. The generator is the ultrasonic power supply that converts electrical energy into an ultrasonic frequency of 30 kHz. The transducer transforms high-frequency electrical signals into kinetic energy (wave motion). The acoustic unit consists of two parts: a booster and a sonotrode. The former modifies the incoming wave from the transducer by amplifying or reducing the wave amplitude, while the sonotrode transfers the vibrational energy to the raw polymer, causing a temperature rise in the material. Finally, the plunger provides the pressure to move the melted material to the mold, where the final specimen with the desired shape is obtained after a fast-cooling process in the mold. Figure 1a shows the main stages of the ultrasonic process; the plasticizer chamber is fed with the polymer, and ultrasonic vibration is transmitted from the sonotrode to the material. The plunger presses the material (Figure 1b), and it flows through the feeding channel filling, the mold that has been heated to a specific temperature (Figure 1c).

For this work, the parameters of amplitude and processing time were varied to study the behavior of iPP in the UMIM process. The wave amplitude causes the polymer to change its physical phase. When combined with time exposure, the polymer can melt and the cavity is filled. The values considered for both parameters were varied based on previously reported studies [1]. The selected values for the amplitude were 80%, 90%, and 100% of the maximum amplitude allowed by the machine, while the times selected were 3, 4, 5, and 6 s. The temperature of the mold and the cooling time were set at 50 °C and 5 s, respectively.

For all the experiments, the injection distance was set to 16 mm (see Figure 1b). This distance was divided in five steps, each one corresponding to a distance of 3 mm. In fact, for safety reasons, the plunger never touches the sonotrode, but remains at a distance of 1 mm at the end of the injection process. The displacement rate of the plunger in each of the five steps is an inset value that is introduced to the machine. For a better understanding of the operation of the UMIM machine, Figure 1d show the inverse of the velocity (in s/mm) set for every step of the four different times used in the present study. In other words, each value in the bar graphs represents the time needed for a displacement of 1 mm. The longer the value, the slower the displacement of the plunger. It can be considered that the first three steps are mainly responsible for melting the polymer, while in the last two the injection and filling of the mold is carried out. In Figure 1d, it can be noticed that the velocity of the plunger in the last two steps remained almost constant, and the differences between the selected times, i.e., 3, 4, 5, and 6 s, are mainly based on the different speeds of the plunger in the first three steps.

### 2.3. Materials Characterization

Differential scanning calorimetry (DSC) was performed using a Discovery model calorimeter from TA Instruments. Samples of around 5 mg were weighed with a precision balance and encapsulated in aluminum pans of known mass. An identical empty pan was used as a reference. Nitrogen was purged at a rate of 50 mL/min. Heating–cooling–heating cycles in the range of −40 to 200 °C were applied with heating and cooling rates of 10 °C/min. The degree of crystallinity of the samples was calculated from the endothermic peak shown in the first heating run, considering an integration range of 50–180 °C and a heat of fusion of 100% crystalline polypropylene of 170 J/g [12].

Small and wide-angle X-ray scattering (SWAXS) measurements were carried out in a SAXSess mc^2^ system from Anton Paar, equipped with a Cu Kα X-ray source with a 0.1542 nm wavelength. Imaging plates were used as an X-ray detector. WAXS 2D patterns were obtained from 0 to 180 in steps of 18 with respect to the orientation direction. The image plate detectors were read out by a Cyclone^®^ Plus Storage Phosphor System (Perkin Elmer, Akron, OH, USA). The collection time was 10 min. WAXS 2D patterns of iPP microinjected probes were obtained under the different ultrasound conditions. All 2D WAXS patterns were treated through the Fraser method and then separated into isotropic and anisotropic fractions, using a previously described image analysis method [13].

The molecular weight of the iPP was measured with an Agilent PL-GPC 220 gel permeation chromatograph equipped with three Agilent MIXED-B LS PL Styragel columns, as well as refractive index and viscometer detectors. The analyses were carried out at 140 °C, using 1,2,4-trichlorobencene as the mobile phase at a flow rate of 1 mL/min. The molecular weight was calculated according to the elution time, which was calibrated using narrow polystyrene standards.

Fourier-transform infrared spectroscopy (FTIR) was carried out on the specimens using a PerkinElmer Frontier spectrometer in the attenuated total reflectance mode (ATR). Spectra were recorded in the spectral range of 4000–380 cm^−1^, with 16 scans and a spectral resolution of 4 cm^−1^.

## 3. Results and Discussion

**Figure 2:**
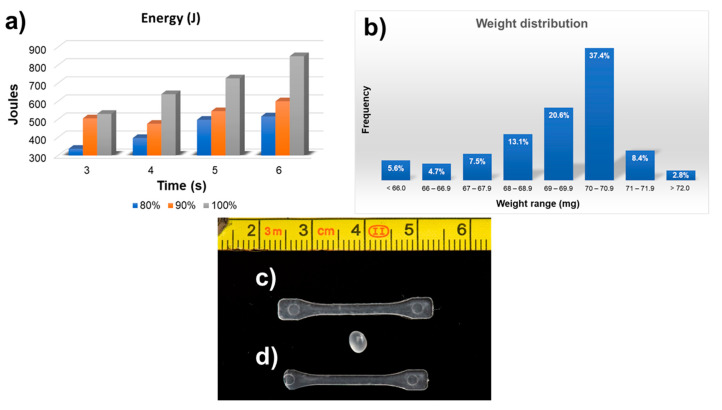
(**a**) Average energy from values displayed by the UMIM machine for each amplitude and time and (**b**) histogram of weight distribution for all the specimens fabricated in the study. Examples of (**c**) a “complete specimen” and (**d**) an “incomplete specimen”. A pellet of raw iPP is placed in between the specimens to visualize the size change after the UMIM processing.

A first analysis of the manufacturing process by UMIM was carried out from the energy values generated by the machine for each of the samples manufactured. The average energy of each amplitude and time values are shown in Figure 2a. The observed trend is the expected one, where the energy increases as the amplitude and time increase. However, it should be noted that the increase in energy is more noticeable when it passes to an amplitude of 100% in times 4 s and longer. That is, the increase in energy when going from 80% to 90% amplitude is not as great as that occurring when going from 90% to 100% amplitude.

The first analyses on the specimens obtained by UMIM were generated from the weight of all the specimens manufactured, to determine a range that could be considered as the ideal weight of the specimens, that is, a weight that can be considered to have high repeatability and thus can be linked with a classification of specimens that have been manufactured correctly. For this, all the specimens obtained in all the experiments (107 specimens in total) were weighed, and from these results the weight distribution histogram shown in Figure 2b was elaborated. This histogram shows that most specimens have a weight between 69 and 70.9 milligrams (58% in sum), specifying that most of the manufactured specimens have a weight within the range of 70–70.9 mg. In fact, the mode of all weight data turned out to be 70.3 mg. In this way, it would be fair to take such a value as the ideal value of a correctly manufactured specimen, considering a variation of 0.1%, as this would place the weight variation within the range where most of the specimens were situated, i.e., 70.3 ± 0.7 mg.

The dumbbell-shaped specimens fabricated by UMIM were analyzed optically in parallel to the weight measurements to classify and distinguish between the fully formed specimens and the incomplete ones. This classification helped to determine which combination of the parameters that were varied, i.e., amplitude and time, generated the conditions to obtain the largest number of fully formed specimens, with at least 12 pieces manufactured continuously and uninterruptedly. Figure 1 shows two specimens, one as an example of a complete specimen (Figure 2c), and one to exemplify an incomplete specimen (Figure 2d). 

The UMIM technique involves the application of ultrasound, which can generate the chain scission of the polymer. As explained by Paulusse and Sijbesma [14], the collapse of cavitation bubbles induces strong shear gradients in addition to hot spots. The hot spots have a very local character, along with the fact that only a few bubbles are formed to implode less violently compared to those of a Newtonian fluid. On the other hand, the mechanical effects, including those explained by Espinoza-Gonzalez [7], occur over much larger distances (micrometers). Only small molecules are translated by these forces, but the effect of the gradient on polymers is much larger and results, in the case of polypropylene, in a chain scission of covalent bonds. The polymer scission in UMIM is a disadvantage because, in fact, the break of molecules is a degradation mechanism of polymers that can affect some material properties if it occurs. Nonetheless, it should be emphasized that the manufacturing process using UMIM requires only a few seconds of exposure to ultrasound. GPC was used to evaluate possible polymer chain breaking in the dumbbell-shaped parts manufactured by UMIM.

**Figure 3:**
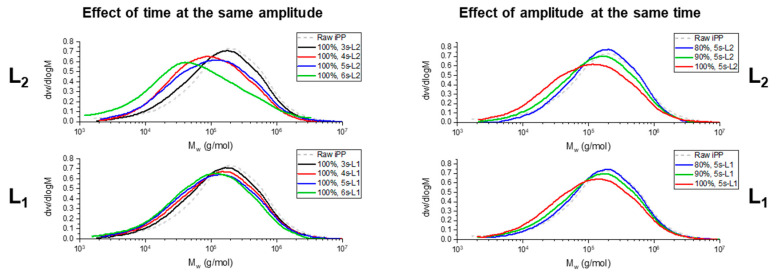
Graphs from GPC to assess the changes in the molecular weight after the UMIM process. On the left, the comparison for different times at the same amplitude (100%). On the right, the comparison for different amplitude at the same time (5 s). L1 corresponds to the side of the specimen closer to the sonotrode, and L2 corresponds to the opposite side.

The GPC results in Figure 3 are shown to observe the effect of the amplitude and time in the change in molecular weight distribution. The analysis was carried out on the two extreme sides of the specimens (L1 and L2), according to the scheme of Figure 1c. The left graphs in Figure 3 compare the effect of time at the same amplitude (100%). Here it can be observed that, as the time increases, the M_w_ distribution is shifted towards a lower molecular weight, indicating greater chain scission, as expected. As the ultrasound exposure time increases, there is a greater probability of the polymer chains breaking. There is also a significant difference between L1 and L2. On the side of the specimen closer to the sonotrode (L1), there is a more subtle molecular weight reduction than on the opposite extreme side, where the difference in M_w_ distribution between the raw polymer and the specimen fabricated at 6 s is noticeable. The graphs on the right in Figure 3 show the effect of the amplitude for the same time (5 s). It can be seen that, as the amplitude increases from 80% to 100%, there is more chain breaking. On the other hand, both sides measured, L1 and L2, show practically the same behavior. That is, the amplitude does not generate a significant difference in the breaking of chains along the same specimen manufactured at a specific amplitude. Table 1 shows a summary of the molecular weight (Mw) and the polydispersity index (PDI) of the samples shown in Figure 3.

**Figure 4:**
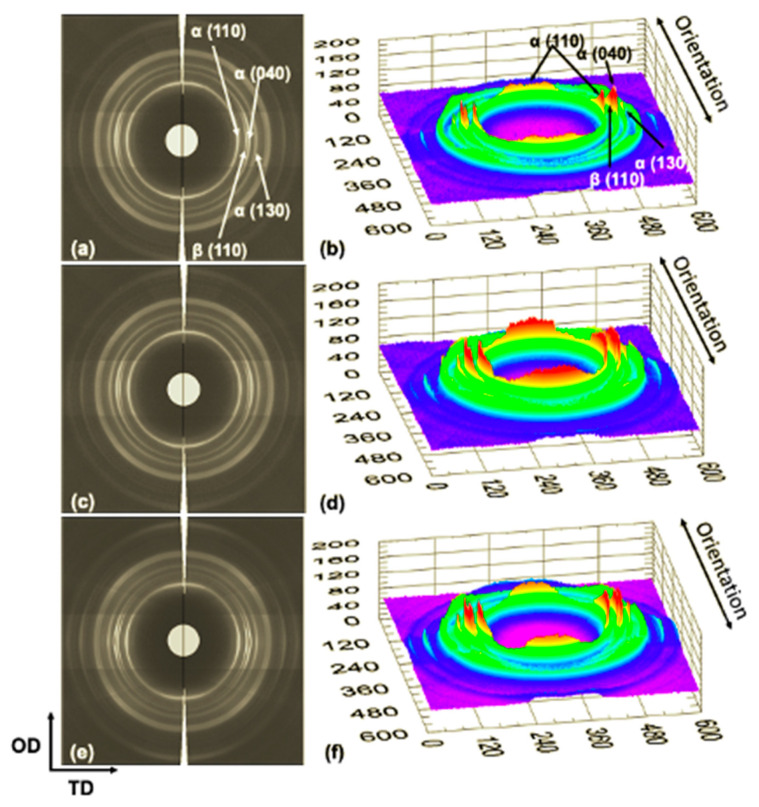
2D and 3D WAXS patterns of the iPP probes injected at constant amplitude (80%) and different times: (**a**,**b**) 3 s, (**c**,**d**) 5 s, and (**e**,**f**) 6 s. The primary crystalline reflections of the α crystalline phase: (110), (040), and (130), and the β crystalline phase (300) of iPP are also shown.

The morphological changes in the crystalline structure generated on the specimens manufactured by the UMIM process were studied by analyzing 2D WAXS images, corrected by the Fraser procedure [13,15,16], as well as through its 3D projection. Firstly, Figure 4 shows the WAXS patterns in 2D (a, c, e) for the samples injected at a constant amplitude of 80% and three different ultrasonic treatment times (3 s, 5 s, and 6 s). In this Figure, it is observed that from the specimen with the lowest treatment time (3 s), a scattering pattern with high anisotropy was generated, given by the preferential orientation in the direction transverse to the orientation direction, mainly in the planes (110), (040), and (130) of the crystal structure of iPP, as well as in plane (300) of the β crystalline habit.

**Figure 5:**
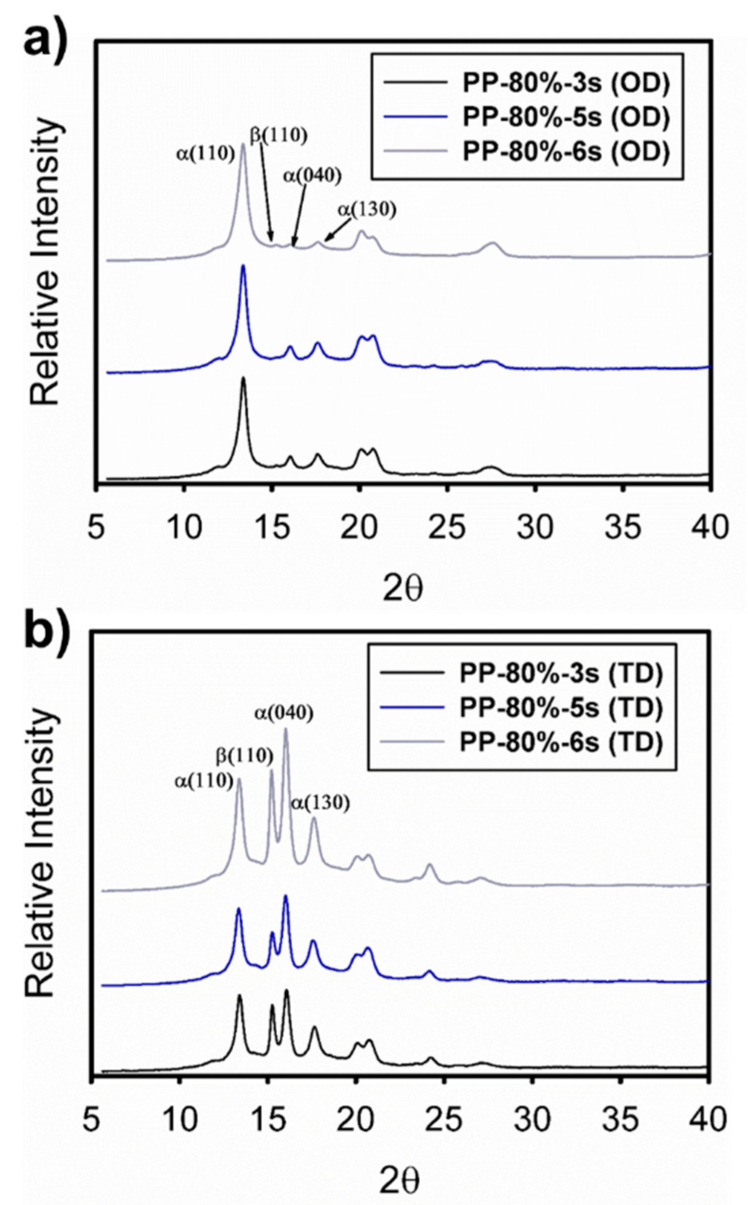
1D WAXS patterns of the iPP specimens injected at constant amplitude (80%) and different times (3 s, 5 s, and 6 s) taken at different orientation directions: (**a**) orientation direction and (**b**) perpendicular or transverse direction. In addition, the primary crystalline reflections of the α crystalline phase: (110), (040), and (130), and the β crystalline phase (300) of iPP are also shown.

This effect is observed in greater detail for the 3D projections (Figure 4b). This behavior in specimens injected under the UMIM process was previously reported and was attributed to the high flow induced by the injection process of UMIM, mainly at higher injection speeds [9]. Furthermore, it has been previously reported [17] that in microinjected parts, the formation of the β crystalline structure occurs mainly in the surface layer of the specimens, while the α phase is mainly in the center of the probe. Additionally, the crystalline orientation of the injected specimen was higher with the lowest treatment time (3 s), decreasing after 5 s, and posteriorly a greater orientation of the α and β crystals was again generated at 6 s. However, the crystalline plane (300) of β crystals decreased with increasing treatment time. This effect is noticeable in the 1D WAXS patterns obtained from integrating the 2D images in the direction transverse to the injection direction of the specimens (see Figure 5).

**Figure 6:**
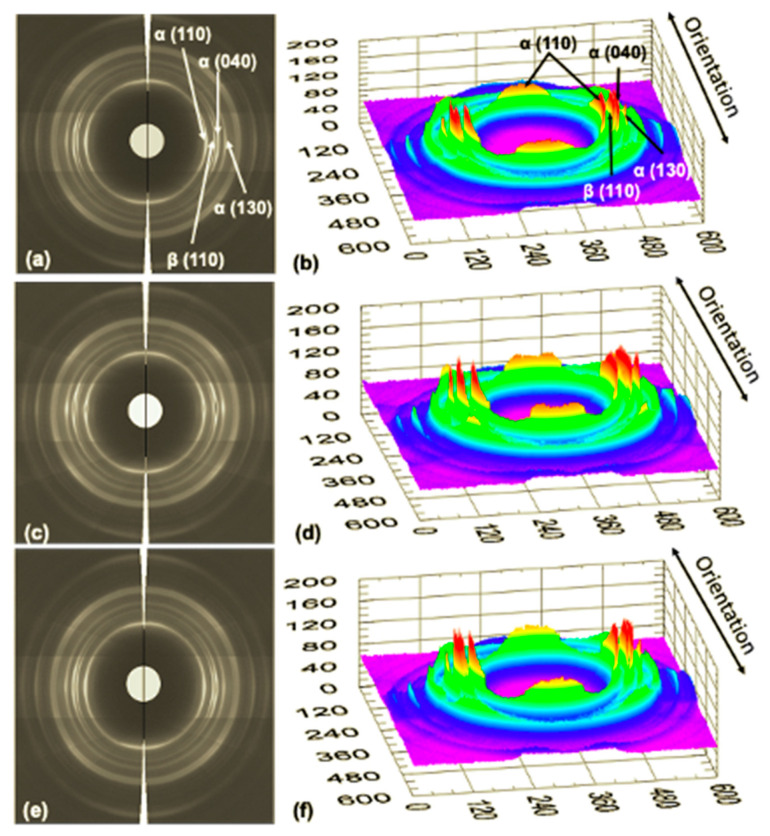
2D and 3D WAXS patterns of the iPP probes injected at constant ultrasound time (6 s) and different amplitudes: (**a**,**b**) 80%, (**c**,**d**) 90%, and (**e**,**f**) 100%. The primary crystalline reflections of the α crystalline phase: (110), (040), and (130), and β crystalline phase (110) of iPP are also shown.

**Figure 7:**
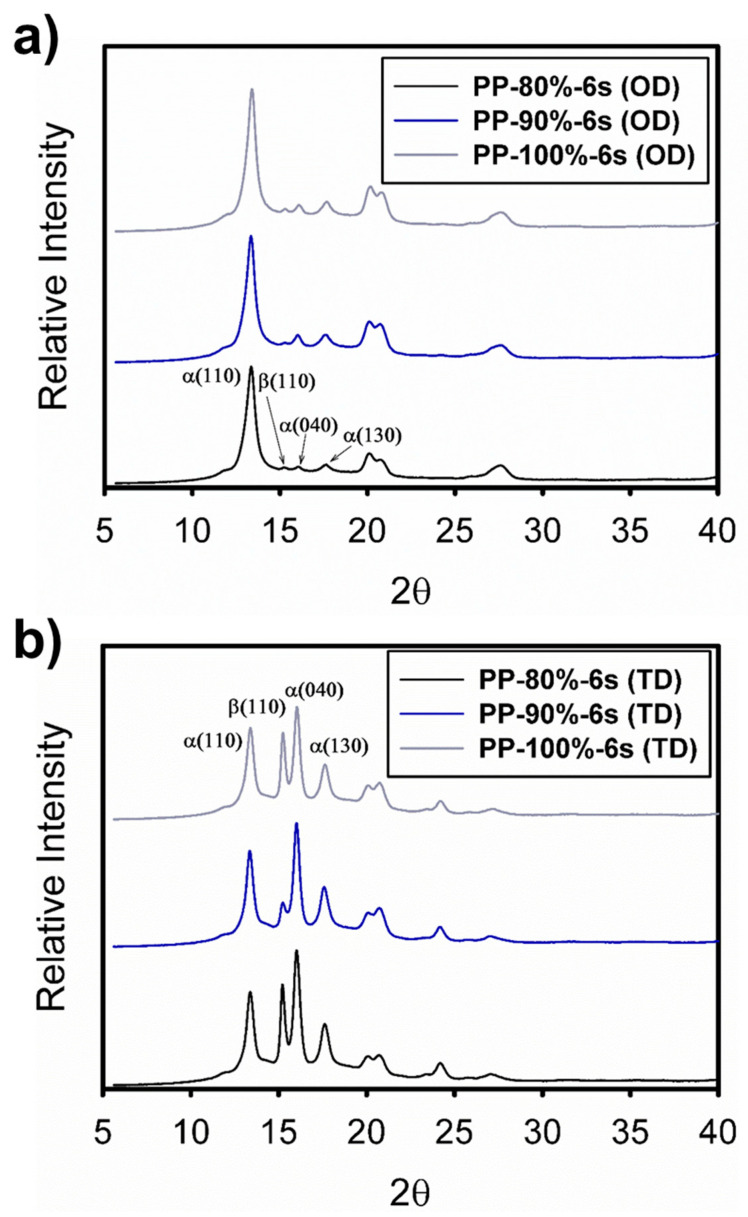
1D WAXS patterns of the iPP probes injected at constant ultrasound time (6 s) and different amplitudes (80%, 90%, and 100%) taken at different orientation directions: (**a**) orientation direction and (**b**) perpendicular or transverse direction directable (110), (040), and (130), and β crystalline phase (300) of iPP are also shown.

On the other hand, Figure 6 shows the effect of the ultrasonic wave’s amplitude on the morphology and structure of the specimens injected using the UMIM process. This figure shows the patterns in 2D and 3D for the specimens injected under the three amplitudes (80%, 90%, and 100%) with 6 s of processing time. In a similar way to that in Figure 4, scattering patterns with high anisotropy are observed given by the preferential orientation in the direction transverse to the orientation direction, mainly in the planes (110), (040), and (130) of the α crystalline structure, as well as in the plane (300) of the β crystalline habit. However, in this case, for the sample with 90% amplitude and 6 s of processing time, the scattering peak of the β phase decreases more drastically, indicating that the formation of this type of crystal in the surface layer is less in this specimen. This behavior is also seen clearly in the 1D WAXS patterns shown in Figure 7.

To determine the relative amount of β phase present in the specimens prepared through the different ultrasound parameters, the standard procedure previously described in the literature was carried out using the Turner–Teller formula [18]:

(1) $$K_{\beta }=\frac{H_{\beta }}{H_{\alpha 1}+H_{\alpha 2}+H_{\alpha 3}+H_{\beta }}$$

where *K_β_* is the relative proportion of beta crystals in the sample; *H_α_*_1_, *H_α_*_2_, and *H_α_*_3_ are the intensities of the diffraction peaks of the crystalline planes (110), (040), and (130) of the α phase, respectively; and *H_β_* is the intensity of the crystalline plane (110) of the β phase. Table 2 shows the proportion of β phase present in the analyzed specimens. This analysis was realized from the 1D WAXS patterns obtained at the TD direction, and the results corroborate the qualitative observations in the 1D patterns, where a decrease in the plane (110) of the crystalline phase β was observed for the PP-80%-5 s and PP-90%-6 s samples.

It has been previously reported that the isotactic polypropylene microinjection process generates a significant presence of polymorphism (α and β phases) oriented on the walls of the specimens [9,17,19]. The decrease of the β phase in the external wall of the specimens has been related to changes in the polymer viscosity generated by the decrease in the molecular weight of polypropylene [20]. In the present work, as reported previously, it was observed that by increasing the time of the ultrasound treatment, keeping the amplitude constant, there was a significant decrease in molecular weight at 5 s and 6 s. Similar to that previously reported in the literature, the decrease in β-phase content in the iPP-80%-5 s specimen may be associated with the decrease in molecular weight; however, although the iPP-80%-6 s sample would have an even lower molecular weight, the percentage of the crystalline phase present in this specimen is higher. This result is contradictory and should be studied in greater detail, mainly due to the differences between both types of crystalline morphology of polypropylene in terms of their mechanical properties [19,21,22].

**Figure 8:**
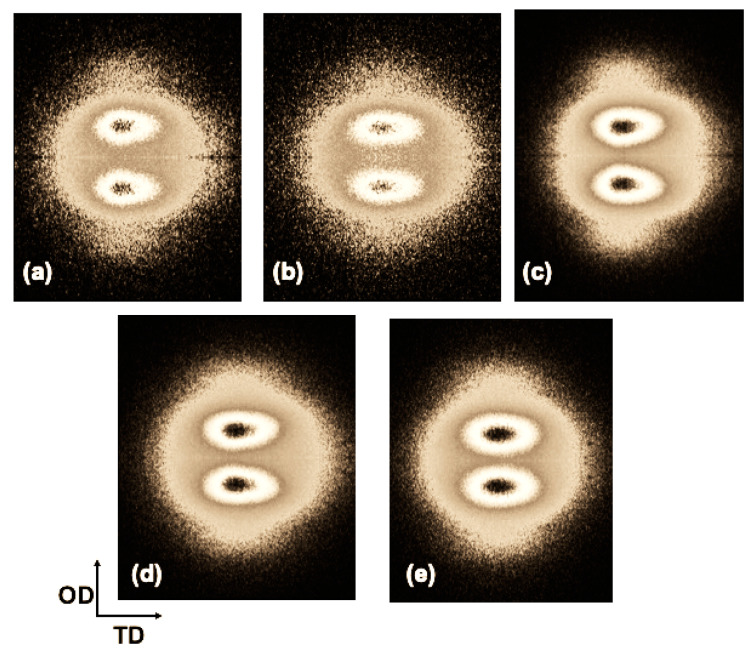
2D SAXS patterns of the iPP probes injected at different conditions: (**a**) PP-80%-3 s, (**b**) PP-80%-5 s, (**c**) PP-80%-6 s, (**d**) PP-90%-6 s, and (**e**) PP-100%-6 s.

On the other hand, 2D SAXS patterns of the specimens injected with a fixed amplitude of 80% and different ultrasound times (3 s, 5 s, and 6 s), as well as those obtained at the same processing time (6 s) but at different amplitudes (80%, 90%, and 100%) are shown in Figure 8. In all cases, an anisotropic scattering pattern is observed, showing two maxima in the meridian region due to oriented lamellar structures (kebabs) [13,23,24,25], induced by the flow during the UMIM process. This pattern was shown with greater intensity when increasing the ultrasound treatment time, mainly for the sample iPP-80%-6 s. However, no significant changes were observed when changing the amplitude to 90% and 100%.

**Figure 9:**
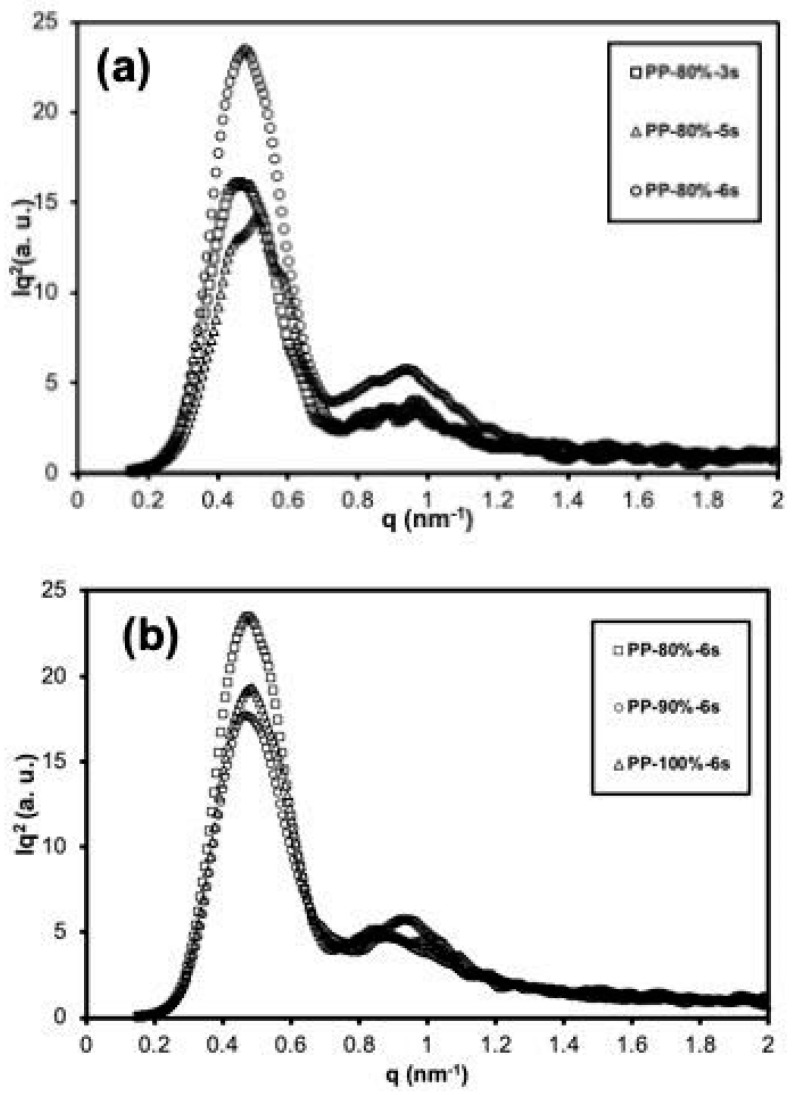
Lorentz corrected 1-D SAXS profiles of iPP specimens injected at two conditions: (**a**) constant amplitude (80%) with different ultrasound times (3 s, 5 s, and 6 s), and (**b**) constant ultrasound time (6 s) and different amplitudes (80%, 90%, and 100%).

Additionally, to determine if there was a change in the thickness of the oriented lamellar structures, the SAXS patterns in 1D were analyzed with Lorentz correction (Figure 9), obtained from the 2D patterns shown in Figure 7. The long-period values were obtained through the equation L = 2π/q_max_. The results of the value of L are shown in Table 3. The results in Figure 6a show that when the processing time was increased at a constant amplitude (80%), the maximum of the scattering signal shifted to higher values, i.e., a small “q”, indicating a greater thickness of the oriented lamellar structures (kebabs) for the sample PP-80%-6 s (L = 13.4 nm) compared to the sample PP-80%-3 s (L = 13.2 nm) and PP-80%-5 s (L = 12.3 nm). It is also clear that the PP-80%-6 s sample dispersion intensity was much higher than for the specimens with a shorter processing time, indicating a greater difference in electron density between the crystalline domain (oriented and non-oriented) and the amorphous domain due to increased orientation.

Conversely, for the specimens obtained at a constant time (6 s) but with a greater amplitude (90% and 100%), the long period practically did not change, and the scattering intensity had a slight decrease.

**Figure 10:**
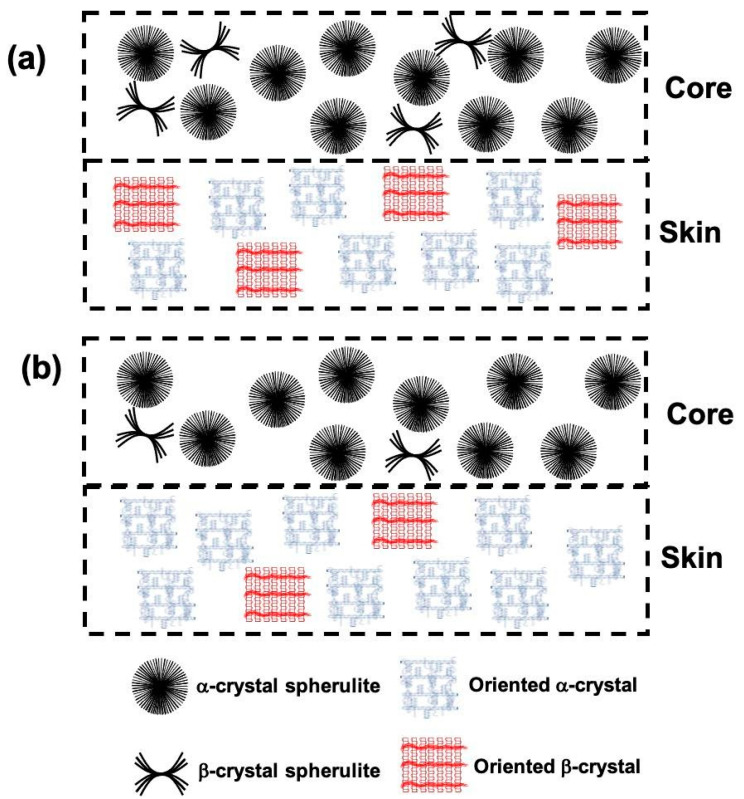
Schematic illustration of crystalline morphology in the skin and core sections of specimens obtained for (**a**) low molecular weight iPP and (**b**) high molecular weight iPP.

The morphological analysis results (SAXS and WAXS) of the specimens injected using UMIM show that the molecular weight of polypropylene played an important role both in the presence of polymorphism (α and β crystals) and in the crystalline orientation, mainly of α crystals. Figure 10 shows an illustrative diagram of crystalline morphology formed in the skin and core regions of the specimens with low molecular weight (Figure 10a) and with high molecular weight (Figure 10b). The low molecular weight is originated by the chain scission in iPP occurring under certain conditions due to the interaction of ultrasound waves with the polymer matrix (see Figure 3). 

**Figure 11:**
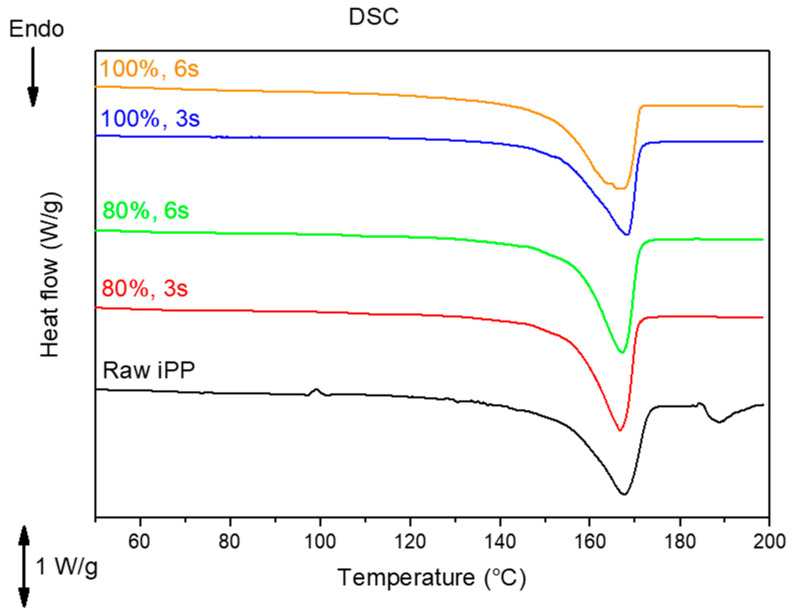
DSC thermograms of specimens fabricated through the UMIM process. For simplicity, only the samples with the lowest and highest of the varied parameters amplitude and time were analyzed.

DSC analysis was carried out using small pieces cut from the central region of the dumbbell-shaped specimens, and the corresponding thermograms are shown in Figure 11. For DSC analysis, only the samples with the lowest and highest amplitude and time were considered to observe the effect of both parameters mainly on the crystallinity. The DSC thermograms show a similar melting point for all samples, i.e., around 167 °C, although, for the sample fabricated with 100% amplitude and 6 s, the endothermic peak was observed to be more widened than that of the rest of the samples. This broadening is pronounced towards lower temperatures, indicating the presence of smaller chains that require a lower temperature to melt. This result agrees with that previously observed by the GPC, where it was determined that with greater amplitude and a longer exposure time to ultrasound, there is greater chain scission.

Regarding crystallinity, Table 4 summarizes the information obtained from the DSC measurements. Here, the higher crystallinity is shown by the samples processed with 80% amplitude. While raw iPP and the samples processed with 100% amplitude have similar crystallinity values of around 50%, the crystallinity of samples processed with 80% amplitude increased to around 64%, i.e., an increase of more than 10%. In this sense, it can be inferred that a lower amplitude allows the formation of more crystalline regions. Considering the previously explained results from the GPC, with an amplitude of 80%, there is less chain scission. However, there is a chain extension due to the ultrasound vibration, combined with the polymer flow in the direction along the mold. Such a combination of processes occurring simultaneously may lead to a better scenario for forming crystalline lamellas in the specimen. This scenario is not expected to occur in samples processed with 100% amplitude, where the polymer chains are extended and scissored, thus reducing the possibility of the formation of large crystalline regions due to short chains.

**Figure 12:**
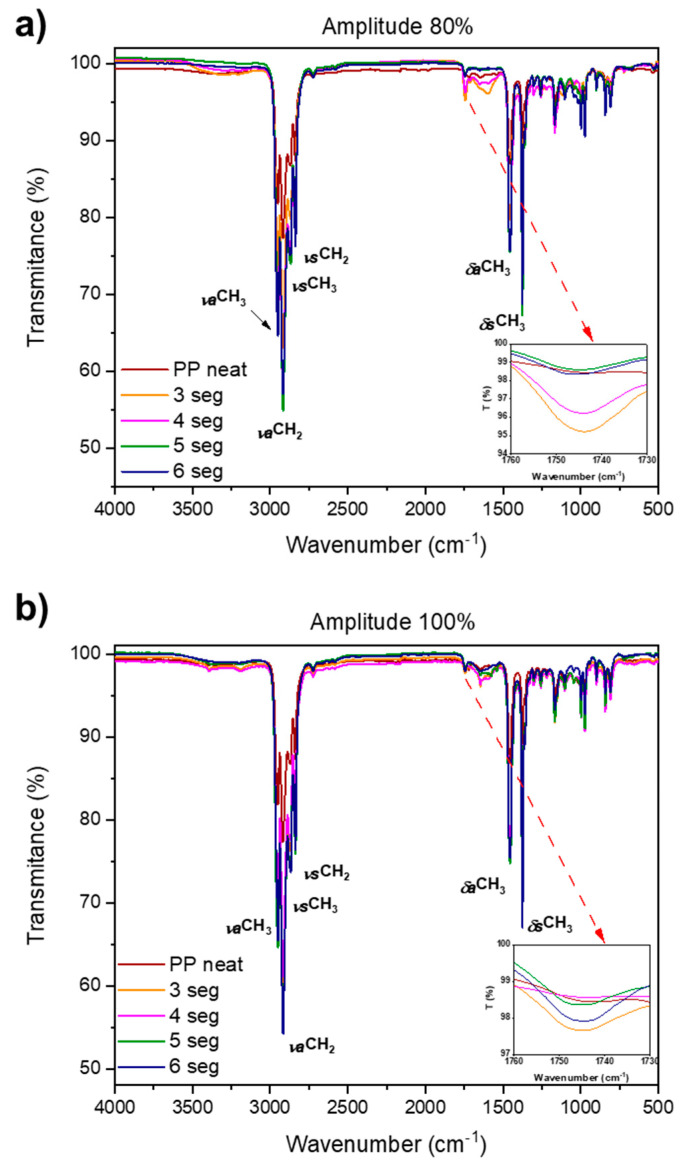
FTIR spectra of specimens fabricated through the UMIM process at different processing times for two selected amplitudes: (**a**) 80%, and (**b**) 100%. The inset graphs demonstrate the oxidation process occurring in the processing carried out at ambient conditions.

An important aspect when analyzing polymers processed at room conditions, specifically without inert conditions as occurs in the UMIM process, is their degradation through oxidation. For this reason, FTIR analyses were carried out on the samples to determine whether functional groups containing oxygen were observed after the fabrication process. The graphs in Figure 12a,b show two different amplitudes, 80% and 100%, respectively, and each graph includes all the processing times. It is worth noting that FTIR analyses were carried out in the center of the dumbbell, that is, the narrowest region of each specimen. The FTIR spectra of the polypropylene samples show the typical peaks for this polymer, that is, those corresponding to the stretching (ν) and deformation (δ) vibrations of the CH_2_ and CH_3_ groups, both symmetric (s) and asymmetric (a). Oxygen groups were tracked because oxidation is the main degradation process of polyolefins. From the FTIR spectra, it can be noted that oxygen is present in the specimens, which is exhibited by the peaks around 1744 and 1600 cm^−1^, attributed to the presence of C=O groups. Also, the samples exhibited a broad peak in the region of 3200–3400 cm^−1^, assigned to the stretching vibration of the O–H group. It is noteworthy that at an amplitude of 100%, the peaks associated to the C=O and O-H groups are narrower compared to the same peaks at 80% amplitude, where they appear broader. This observation can be related to the crystallinity results showed before from the DSC analysis, because narrow peaks in IR spectroscopy are observed when intermolecular interactions are weak, for instance, in the case of diluted samples. In this case, it is not related to a diluted sample, but to a less crystalline polymer where the intermolecular interactions are weak due to a more disordered structure.

The characterization techniques used in this work show clear evidence that chain scission occurs in the iPP during the processing by UMIM, which is very likely to occur in all the polymers that are processed by this technique. Chain scission can be related to the polymorphism observed in the samples, as well as to the plasticization process that may occur inside the chamber, which in turn would allow the polymer to flow into the mold. Although the exact mechanism of plasticization that occurs during the action of ultrasound waves generated by the sonotrode remains unclear, the results presented here suggest that special attention should be paid to the fact that the manufactured parts will present differences in molecular weight and crystallinity compared to those of the raw polymer, as well as a certain degree of chain orientation and oxidation as a consequence of this manufacturing process. By considering this, it can be anticipated that some polymer properties, mainly mechanical, may be affected. More work is required to deepen the knowledge of the UMIM technique, mainly directed at considering the viscoelastic properties of polymers.

## 4. Conclusions

The behavior of iPP processed by the UMIM technique was studied in detail by analyzing the polymer molecular structure. The UMIM technique is a relatively new process with great application potential in the miniaturization of parts. It is also considerably different from conventional polymer processing techniques, so it is of great importance to understand as well as possible the behavior of polymers in this process. This work demonstrates that changes in the molecular structure of iPP occur during UMIM processing, the most remarkable being the chain scission by the action of ultrasound waves, as well as the presence of polymorphism. It is noteworthy that in the UMIM process, which is carried out in a relatively short time and small space, a certain degree of chain orientation is obtained, similar to some conventional polymer processing technologies. The importance of knowing the effect that the various adjustable parameters have within UMIM processing is beneficial because, in this way, the best combination of parameters can be found depending on the polymer to be used or the application desired for the part. In this paper, it was shown that both the amplitude and the processing time directly influence the chain scission; however, time is a more relevant factor for this effect as it is capable of achieving a greater chain scission in different areas of the same specimen.

## Figures and Tables

**Table 1:** Molecular weight (M_w_) and polydispersity index (PDI) of samples fabricated by UMIM. PDI is calculated as M_w_/M_n_.

Sample	M_w_ (g/mol)		PDI	
Raw iPP	351,716		7.118	
**100% of amplitude**				
	**L1**		**L2**	
**Time**	**M_w_ (g/mol)**	**PDI**	**M_w_ (g/mol)**	**PDI**
3 s	301,980	5.559	289,005	5.511
4 s	280,775	6.283	205,118	5.288
5 s	269,195	6.455	252,869	6.762
6 s	220,866	6.311	186,246	10.172
**5 s of processing time**				
	**L1**		**L2**	
**Amplitude**	**M_w_ (g/mol)**	**PDI**	**M_w_ (g/mol)**	**PDI**
80%	322,128	5.03	297,087	3.787
90%	292,838	5.886	273,832	5.134
100%	269,195	6.455	252,869	6.762

**Table 2:** Amount of β-crystal quantified by WAXS for specimens injected by the UMIM process.

Sample	β Crystal (%) by WAXS
PP-80%-3 s	24.4
PP-80%-5 s	20.4
PP-80%-6 s	25.9
PP-90%-6 s	14.1
PP-100%-6 s	25.2

**Table 3:** Scattering vector (q) and long-period (L) values obtained from 1D-SAXS patterns for various UMIM specimens.

Sample	q (nm^−1^)	L (nm)
PP-80%-3 s	0.48	13.2
PP-80%-5 s	0.51	12.3
PP-80%-6 s	0.47	13.4
PP-90%-6 s	0.47	13.4
PP-100%-6 s	0.49	12.9

**Table 4:** Summary of results from DSC. Amplitude affects the crystallinity, and the chain scission is more evident in the onset of the melting temperature (T_m_).

	1st Heating Cycle			
Sample	ΔH	Crystallinity	Onset T_m_	T_m_ (peak)
	(J/g)	(%)	(°C)	(°C)
Raw iPP	87.011	51.2%	154.0	167.9
iPP_80-3	108.81	64.0%	155.7	166.6
iPP_80-6	110.33	64.9%	156.0	167.1
iPP_100-3	85.466	50.3%	154.1	168.3
iPP_100-6	87.553	51.5%	152.1	166.4
